# *Salmonella enterica* Serovar Enteritidis Enterocolitis during Late Stages of Gestation Induces an Adverse Pregnancy Outcome in the Murine Model

**DOI:** 10.1371/journal.pone.0111282

**Published:** 2014-11-03

**Authors:** Mariángeles Noto Llana, Sebastián Hernán Sarnacki, María del Rosario Aya Castañeda, María Carolina Pustovrh, Alejandra Sonia Gartner, Fernanda Roxana Buzzola, María Cristina Cerquetti, Mónica Nancy Giacomodonato

**Affiliations:** 1 Instituto de Investigaciones en Microbiología y Parasitología Médica, Universidad de Buenos Aires-Consejo Nacional de Investigaciones Científicas y Técnicas (IMPaM, UBA-CONICET), Buenos Aires, Argentina; 2 Departamento de Morfología, Universidad del Valle, Cali, Colombia; Medical Faculty, Otto-von-Guericke University Magdeburg, Medical Faculty, Germany

## Abstract

Foodborne diseases caused by *Salmonella enterica* serovar Enteritidis (*S.* Enteritidis) are a significant health problem. Pregnancy, state of immunological tolerance, is a predisposing condition for the development of infections with intracellular pathogens. *Salmonella* species can cause pregnancy complications such as chorioamnionitis, transplacental fetal infection, pre term labor, abortions, neonatal and maternal septicemia. However, the specific mechanisms by which *Salmonella* infections trigger these alterations are not clear. In the present work, using a self-limiting enterocolitis murine model, we show that the ingestion of a low dose of *S.* Enteritidis at late stages of pregnancy (day 15 of gestation) is sufficient to induce massive maternal infection. We found that *Salmonella* infection leads to 40% of pre term delivery, 33% of abortion and fetal growth restriction. Placental dysfunction during *S.* Enteritidis enterocolitis was confirmed through cellular infiltration and hypoxia markers (MPO activity and COX-1 and COX-2 expression, respectively). Apoptosis in placental tissue due to *Salmonella* infection was also evident at day 18 of gestation when investigated by morphometric procedure, DNA fragmentation and Fas/FasL expression. Also, the expression of IFN-γ, TNF-α, IL-17 and IL-10 was up regulated in response to *Salmonella* not only in placenta, but also in amniotic fluid and maternal serum. Altogether, our results demonstrate that *S.* Enteritidis enterocolitis during late stages of gestation causes detrimental effect on pregnancy outcome.

## Introduction

Foodborne illness accounts for substantial morbidity and mortality in the United States and has a major public health impact. In 2011 Centers for Disease Control and Prevention estimate that each year roughly 1 in 6 Americans (or 48 million people) get sick, 128,000 are hospitalized, and 3,000 die of foodborne diseases (http://www.cdc.gov/foodborneburden/2011-foodborne-estimates.html). *Salmonella* is the most common bacterial cause of confirmed, single-etiology outbreaks and illnesses. Among the confirmed *Salmonella* outbreaks with a serotype reported, Enteritidis is the most common, followed by Typhimurium [1]. The severity and the outcome of a foodborne *Salmonella* infection depends on the “virulence” of the bacteria, on the infectious dose as well as on the genetic makeup and immunological status of the host [2]. A key component of the response to emerging infections, such as foodborne illness, is consideration of special populations [3]. Young, elderly, pregnant, and HIV-infected individuals form the high-risk groups for *Salmonella* infections [4].

Pregnancy is a condition where the immune system is faced with the situation of not rejecting the foreign paternal antigens of the fetus while protecting self and the fetus from invading pathogens. The immune system overcomes this dilemma by altering the Th1/Th2 cytokine levels to favor Th2 cytokines [5]. This protects the fetus from the Th1 mediated immune rejection at the fetal-maternal interface but at the same time makes pregnant women susceptible to infections where immunity is Th1-dependent like in malaria [6], leishmaniasis [7], toxoplasmosis, leptospirosis [8] and salmonellosis [9]. *Salmonella* species can cause pregnancy complications such as chorioamnionitis, transplacental fetal infection, pre term labor, abortions, neonatal and maternal septicemia [10], [11]. The exact mechanisms by which *Salmonella* infection causes complications during pregnancy are not clear. In the present work, using a self-limiting enterocolitis murine model, we were able to identify some of the factors that induce a detrimental effect on pregnancy outcome after a foodborne illness. We show that *S.* Enteritidis infection is exacerbated in pregnant hosts and rapidly proliferates in the placenta leading to pre term delivery and abortion within 3 days post infection. Additionally, we explore apoptosis, inflammation, and hypoxia in placental tissue and inflammatory cytokines in maternal serum, amniotic fluid and placenta during *Salmonella* infection.

## Materials and Methods

### Mice, mating and pregnancy

Six to 8–week old female BALB/c mice were obtained from our vivarium, maintained under standard conditions and provided with food and water *ad libitum*. For mating, male and female mice were caged overnight, and detection of vaginal plugs the following morning was considered day 1 of pregnancy. At the end of each experiment, mice were sacrificed with carbon dioxide. All experimental protocols were approved by the Animal Ethics Committee, University of Buenos Aires.

### Bacteria

Wild-type strain of *Salmonella enterica* serovar Enteritidis #5694 (*S*. Enteritidis) was used to infect mice. Bacteria were cultured in trypticase soy broth at 37°C, 200 cycles per minute, then pelleted by centrifugation and suspended to the appropriate density in saline solution. In all cases the number of bacteria was determined by plating appropriate dilutions on trypticase soy agar plates.

### *S.* Enteritidis infection and generation of enterocolitis

Late term pregnant mice were pretreated at day 14 of gestation with 20 mg of streptomycin (Sigma Aldrich) given intragastrically [12] and 24 h later they received 3–4×10^3^ CFU of *S.* Enteritidis (LD50 is 10^4^ CFU) by the same route. For intragastric infection, 0.2 ml of the bacterial suspension was introduced into the stomach with a 21 G blunt needle on a 1.0 ml plastic syringe. A group of infected pregnant mice was allowed to deliver normally, monitored everyday and put under constant surveillance for signs of parturition and sickness. Uninfected pregnant (with or without streptomycin treatment) mice were included as control and observed similarly. A second group of pregnant mice (infected and controls) were sacrificed 3 days after infection (day 18 of gestation).

### Assessment of bacterial burden in organs

After natural delivery or at day 3 post infection (day 18 of gestation) mice were sacrificed and bacterial loads were analyzed. All Peyer's patches located along the large intestine (6 to 8), intestine, spleen, placenta, uterus and fetuses were removed aseptically from each animal and homogenized in 1.0 ml of sterile saline solution. Thus, the number of CFU/ml equals the number of CFU/organ. Samples were diluted appropriately in saline and plated on *Salmonella*-*Shigella* (SS) agar. Colonies were counted after 24 h of incubation at 37°C.

### Fetal growth restriction (FGR)

Fetuses delivered at term or pre term (naturally or by cesarean) were weighted. FGR was defined as fetuses with weights 2 standard deviations (SD) smaller (i.e., less than 1.421 g) than normal fetal weight (1.543±0.061 g) at 21 days of gestation and smaller (i.e., less than 1.049 g) than normal fetal weight (1.231±0.091 g) at 18 days of gestation as described Lin et al. [13].

### Myeloperoxidase Assay

The myeloperoxidase (MPO) assay was performed as described earlier [14]. In brief, placental samples were suspended in 1.0 ml of 50 mM potassium phosphate buffer (pH 6.0) containing 0.5% hexadecyltrimethylammonium bromide (Sigma, Missouri, USA) followed by sonication on ice for 15 s. Suspensions were then freeze-thawed three times and the supernatant was separated from the solid phase by centrifugation at 16,000 g for 20 min. A total of 10 µl of the supernatant was mixed with 140 µl of 50 mM phosphate buffer (pH 6.0) containing 0.167 mg/ml o-dianisidine dihydrochloride (Sigma) and 0.005% hydrogen peroxide (Sigma). MPO activity was derived from an observed change in absorbance measured by spectrophotometry at 450 nm (Bio-Rad, California, USA) and normalized to the total protein content of the supernatant.

### Quantitative real-time reverse transcriptase-polymerase chain reaction

Total RNA was extracted from tissues using Trizol reagent (Life Technologies, Inc, Carlsbad, CA) at different time points according to the experiment. Total RNA (1 µg per sample) was reverse transcribed with oligo(dT) as primer using Expand Reverse Transcriptase (Promega Corporation, Madison, WI) according to the manufacturer's protocol. Quantitative real-time reverse transcriptase-polymerase chain reaction was performed using SyBr Green PCR kit (Applied Biosystems Inc, Foster City, CA) in an Applied Biosystems 7500 sequence detector. Primer sequences are described in Table 1. All samples were analyzed in the same run for 18 s expression for normalization. Polymerase chain reaction parameters were 50°C for 2 min, 94°C for 2 min, and 40 cycles of 94°C for 30 s and 60°C. Quantification of gene expression was calculated using the comparative threshold cycle (Ct) method, normalized to the 18 s control and efficiency of the RT reaction (relative quantity, 2-ΔΔCt). The replicates were then averaged, and fold induction was determined, considering the value in “control” group as 1 [17].

**Table 1:** Primers used for quantitative real-time reverse transcriptase-PCR assays.

mRNA targeted	Sequence (5′→3′)(a)	Ref
COX-1	GTGCTGGGGCAGTGCTGGAG (F)	[15]
	TGGGGCCTGAGTAGCCCGTG (R)	
COX-2	GCTGTACAAGCAGTGGCAAAG (F)	[16]
	GCGTTTGCGGTACTCATTGAGA (R)	
TNF-α	ATGAGCACAGAAAGCATGATC (F)	[17]
	TACAGGCTTGTCACTCGAATT (R)	
IL-17	GCTCCAGAAGGCCCTCAGA (F)	[18]
	AGCTTTCCCTCCGCATTGA (R)	
IL-10	CCAAGCCTTATCGGAAATGA (F)	[17]
	TTTTCACAGGGGAGAAATCG (R)	
IFN-γ	ACAATGAACGCTACACACTGCAT (F)	[19]
	TGGCAGTAACAGCCAGAAACA (R)	
FAS	ATGCACACTCTGCGATGAAG (F)	[20]
	CAGTGTTCACAGCCAGGAGA (R)	
FAS-L	ATTGAAGAAGACACCTTTACACTCATT (F)	[21]
	TGTCTCAAAAACGGCCTCTGT (R)	
18s rRNA	AACACGGGAAACCTCACCC(F)	[17]
	CCACCAACTAAGAACGGCCA (R)	

Primers were purchased from Invitrogen Inc. and were designed according to the DNA sequence information available for *Mus musculus* (*M. musculus* blast server BLAST Server Database at www.sanger.ac.uk).

(a) F, forward primer; R, reverse primer.

### Cytokine Analysis by ELISA

For TNF-α, IL-17, IFN-γ and IL-10 determinations, placental, amniotic fluid and maternal blood samples were obtained 3 days after oral inoculation with *S.* Enteritidis from pregnant mice. Uninfected pregnant hosts were used as controls. Briefly, placentas (pooled per mouse) were cut into pieces and homogenized in 1.0 ml of PBS plus 1% BSA. Tissue homogenates were subjected to centrifugation (12.000 rpm, 1 min) to pellet all cell debris prior to concentration using an Amicon Ultra-4 Centrifugal Filter Unit (Merck Millipore). Supernatants were stored at −20°C until further use. Analyses were conducted using commercially available enzyme-linked immunosorbent assay (ELISA) kits: TNF-α and IL-17 (R&D Systems, Minneapolis, MN), IFN-gamma and IL-10 (OptEIA BD Pharmingen, San Diego CA) according to manufacturer's instructions. Cytokine levels were expressed as picogram per ml (pg/ml).

### Percent apoptotic tissue

Apoptotic cells were counted in hematoxylin-eosin-stained sections using the morphometric procedure described by Zhou et al [22]. Briefly, sections were examined under light microscopy. Cells were scored as apoptotic if they exhibited cellular shrinkage with concurrent cytoplasmic eosinophilia, nuclear pyknosis, and fragmentation with associated apoptotic bodies. Digitized images of randomly selected fields were obtained and characterized morphometrically in terms of percent apoptotic lymphoid tissue. Images were captured and analyzed using a double-density 25/125-point grid. The tissue beneath each grid point was categorized as normal lymphoid tissue, apoptotic lymphoid tissue, or other (extracellular space, blood vessels, etc.). The number of points in each category was totaled individually for every animal. The percentage of apoptotic tissue for each animal was calculated from the area of lymphoid tissue: %area _(apoptotic)_  =  (area _(apoptotic)_/[area _(normal)_ + area _(apoptotic)_]) ×100.

### Cell death detection by ELISA

DNA fragmentation was assessed by using a sandwich ELISA (cell death detection ELISA kit; Boehringer Mannheim, Indianapolis, Ind.). Briefly, single-cell suspensions were prepared by smashing placentas with a glass plunger against a fine stainless steel wire net submerged in ice-cold phosphate-buffered saline (PBS). Approximately 3×10^4^ cells were lysed, and the cytosolic oligonucleosomes were quantified using a biotin-coupled mouse monoclonal antihistone antibody as the capturing antibody, peroxidase-conjugated mouse monoclonal anti-DNA antibody as the detecting antibody, and ABTS (2,2′-azino-di[3-ethylbenzthiazoline]- sulfonate) as the developing reagent. The relative increase in nucleosomes in the cytoplasm was expressed as an enrichment factor, calculated as the ratio of specific absorbance in lysates from treated mice compared with that in control animals, as described by the manufacturer.

### Statistical analysis

Data were analyzed for statistical significance using a nonparametric Mann-Whitney test. Statistical analysis was performed using the software program Prism 4.0 (GraphPad Software, San Diego, CA, USA). P values less than 0.05 were considered statistically significant.

## Results

### Adverse pregnancy outcome after *S*. Enteritidis enterocolitis

In this work we use an enterocolitis murine model for studying the effect of *Salmonella* infection during pregnancy at late stages of gestation (day 15). Groups of 20 pregnant mice were pretreated with streptomycin and afterwards infected intragastrically with 10^3^ CFU of *S.* Enteritidis. Animals were monitored everyday and put under constant surveillance for signs of parturition and sickness. Groups of uninfected pregnant mice (with or without streptomycin treatment) were included as controls and observed similarly. Results are depicted in Table 2. All pregnant mice (infected and uninfected) survived until delivery. Fifteen percent (3/20) of infected mothers showed some sign of disease (mainly consisting of rough hair coat and lethargy). No pregnancy complications were observed in uninfected mothers treated with streptomycin. Therefore, this control group was not further included in the experiments. On the contrary, infection with *S.* Enteritidis during late stages of pregnancy induced 40% of premature labor (8 out of 20 infected pregnant mice) and 33% of abortion (38 out of 116 total pups). It is worth noting that the majority of the pre term deliveries took place at day 18 of gestation.

**Table 2:** Pregnancy outcome after *S.* Enteritidis enterocolitis.

Dose of streptomycin (mg/mice)	Dose CFU/mice	Survival(a)	Premature labor(b)	Abortion(c)
20	10^3^	20/20	8/20	38/116
0	0	20/20	0/20	0/134
20	0	5/5	0/5	0/31

Late term pregnant mice were pretreated with 20 mg of streptomycin (day 14 of gestation) before intragastric infection with 3–4×10^3^ CFU of *S.* Enteritidis. Two groups of uninfected pregnant mice (with or without streptomycin treatment) were included as controls. Animals were allowed to deliver normally, monitored everyday and put under constant surveillance for signs of parturition.

(a) Number of survivors/total number of mothers.

(b) Number of premature labors/total number of labors.

(c) Number of aborted pups/total number of pups.

Bacterial burden was analyzed in placenta and pups delivered pre term and at term by infected mothers. As shown in Table 3, pre term delivered placentas present significantly higher *Salmonella* colonization compared with the at term group (median: 3×10^6^ CFU/placenta vs. 7×10^2^ CFU/placenta, respectively; p<0.01). In the same way, pups delivered prematurely were highly colonized (Table 3). There was a significant difference in the bacterial burden between pre term pups and those delivered at term (median: 2×10^3^ CFU/pup vs. 4.5×10^1^ CFU/pup, respectively; p<0.05). These results demonstrate that *S.* Enteritidis food borne illness can cause abortion, pre term labor and offspring infection without causing maternal lethal effects.

**Table 3:** Bacterial burden in placenta and pups delivered pre term or at term after *S.* Enteritidis enterocolitis.

	Pre term delivery	At term delivery
**Placenta** (a) **CFU/organ**	3×10^6^ * (10^5^–5×10^6^)	7×10^2^ (10^1^–10^3^)
**Pups** (a) **CFU/pup**	2×10^3^ ‡ (10^2^–4×10^3^)	4.5×10^1^ (2–6×10^1^)

A group of 20 pregnant mice received 20 mg of streptomycin 24 h before intragastric infection with 3–4×10^3^ CFU of *S.* Enteritidis (day 15 of gestation). Animals were allowed to deliver normally. Pups and placentas were removed and bacterial loads were analyzed as described in materials and methods.

(a) Median (range).

*p<0.01;

‡ p<0.05 in comparison with the at term delivered group.

### Pregnancy favors *S.* Enteritidis dissemination soon after oral inoculation

Next we decided to investigate the outcome of salmonellosis at late stages of pregnancy. Thus, groups of 15 pregnant mice were infected with *S.* Enteritidis as described above and euthanized at day 18 of gestation. Immediately afterwards fetuses were removed aseptically by cesarean operation. The presence of *Salmonella* was assessed in maternal Peyer's patches, spleen, intestine, uterus, placenta and fetuses. Groups of non pregnant mice were infected as described above and included as controls. As shown in Table 4, infection of pregnant mice resulted in a massive maternal organs and fetuses colonization. The bacterial burden was profoundly increased in infected mothers (ca. 10,000-fold higher in Peyer's patches and spleen, and 100-fold higher in the gut) in comparison to non pregnant infected mice (Table 4). The exacerbation of *S.* Enteritidis infection observed in pregnant mice also correlated to a substantial bacterial colonization of the placenta, uterus, and fetuses at day 3 post infection (Table 4). These results demonstrate that pregnancy is a predisposing condition for the development of infections with intracellular pathogens, like *S.* Enteritidis.

**Table 4:** Organ colonization in pregnant and non pregnant hosts after *S.* Enteritidis enterocolitis.

Mice	Dose CFU/mice	Peyer's patches(a) CFU/organ	Spleen(a) CFU/organ	Intestine(a) CFU/organ	Uterus(a) CFU/organ	Placenta(a) CFU/organ	Fetus(a) CFU/fetus
**Pregnant**	10^3^	5,8×10^5^ *	6,4×10^5^ *	4,0×10^4^ ‡	4,9×10^5^	2×10^5^	7,6×10^2^
		(10^4^–8×10^5^)	(10^4^–10^6^)	(10^3^–7×10^4^)	(10^5^–5×10^6^)	(10^5^–5 ×10^6^)	(10^5^–5×10^6^)
**Non pregnant**	10^3^	6×10^1^	7×10^1^	1.5×10^2^	-	-	-
		(2×10^1^–8×10^1^)	(2×10^1^–9×10^1^)	(2×10^1^–3×10^2^)			

Groups of 15 pregnant and non pregnant mice received 20 mg of streptomycin 24 h before intragastric infection with 3–4×10^3^ CFU of *S.* Enteritidis (day 15 of gestation in case of pregnant hosts). At day 3 after infection mice were sacrificed and bacterial loads were analyzed as described in materials and methods.

(a) Median (range).

*p<0.01;

‡ p<0.05 in comparison with the non pregnant infected.

### Enterocolitis by *S.* Enteritidis induces inflammatory cytokine response in placenta, amniotic fluid and maternal serum

**Figure 1:**
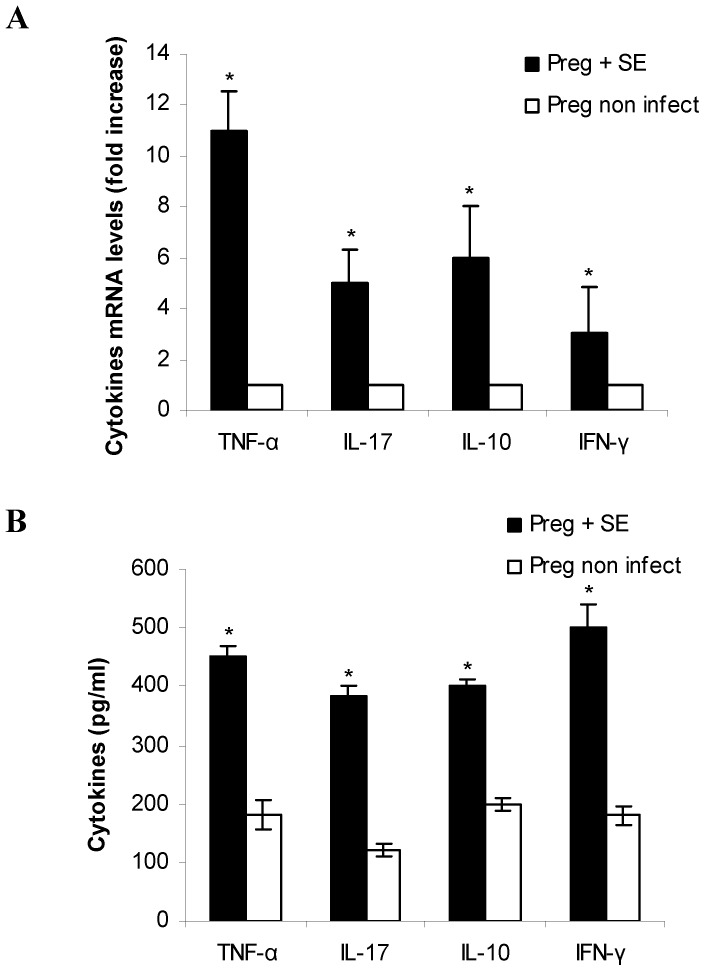
Placental cytokine expression after *S.* Enteritidis enterocolitis. Pregnant mice received 20 mg of streptomycin 24 h before intragastric infection with 3–4×10^3^ CFU of *S.* Enteritidis (day 15 of gestation). A group of non infected pregnant mice was included as control. Placentas (healthy and infected) were obtained on day 18 of gestation. **A.** The relative cytokine mRNA expression was determined by qPCR and related to mRNA levels in non infected control, set as 1. **B.** Cytokines levels were measured in placental homogenates by ELISA as described in materials and methods. Placentas from each individual mouse were pooled. Five animals per group were analyzed. Results are expressed as mean +/− SD. *p<0.05 respect to uninfected control placentas. Representative data from 3 independent experiments.

**Figure 2:**
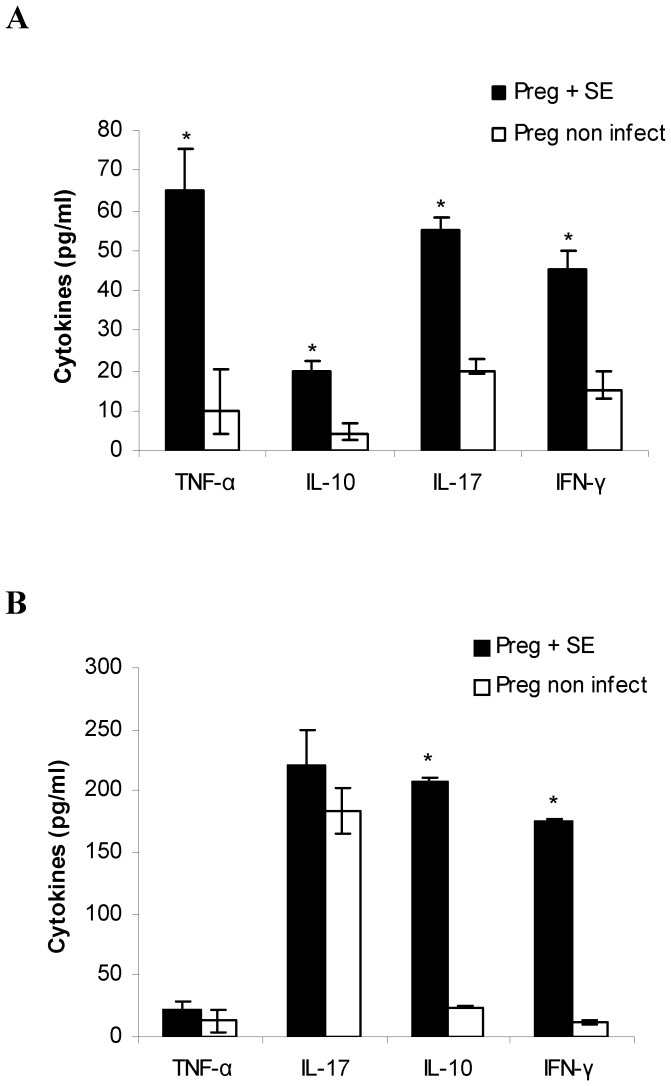
Cytokine expression in amniotic fluid and maternal serum after *S.* Enteritidis enterocolitis. Pregnant mice received 20 mg of streptomycin 24 h before intragastric infection with 3–4×10^3^ CFU of *S.* Enteritidis (day 15 of gestation). A group of non infected pregnant mice was included as control. Samples were obtained on day 18 of gestation. Cytokines levels were measured by ELISA **A.** in pooled amniotic fluid from individual infected mothers and **B.** in maternal serum. Five animals per group were analyzed. Results are expressed as mean +/− SD. *p<0.05 respect to uninfected control. Representative data from 3 independent experiments.

During pregnancy, cytokine ratios are altered to facilitate allograft survival. Increase in Th1 cytokine levels can lead to abortion and pre term labor [5]. Because our results showed that enterocolitis by *S.* Enteritidis causes an adverse pregnancy outcome, we next investigated whether *S.* Enteritidis infection induces cytokine response in the placental tissue, amniotic fluid and maternal serum. To this purpose infected and non infected pregnant mothers were sacrificed at day 3 post infection (day 18 of gestation) and samples were removed as described in materials and methods. Inflammatory (TNF-α, IL-17, IFN-γ) and anti-inflammatory (IL-10) cytokines were determined by qPCR and ELISA. We found that *S.* Enteritidis-infected placentas present a significant increase in the expression of TNF-α, IL-17, IFN-γ and IL-10 compared with uninfected control (Figure 1A). Similar results were obtained by ELISA (Figure 1B). The pattern of cytokines investigated in amniotic fluid is presented in Figure 2A. We found significantly higher levels of TNF-α, IL-17 and IFN-γ in the infected hosts compared to the healthy pregnant ones (p<0.05). IL-10 levels were also elevated in the amniotic fluid of infected pregnant mice compared with non infected mothers (p<0.05) (Figure 2A). Maternal circulating cytokines were also evaluated. We found that, in comparison to non infected mice, *S.* Enteritidis infection evokes increased production of IFN-γ and IL-10 (p<0.05) (Figure 2B). In contrast, circulating IL-17 and TNF-α were similar in both animal groups on day 3 post infection (Figure 2B). In total, the increase in Th1 cytokines observed could explain the deleterious effect of *Salmonella* infection on pregnancy outcome.

### *S.* Enteritidis enterocolitis during late stages of pregnancy results in fetal growth restriction (FGR)

**Figure 3:**
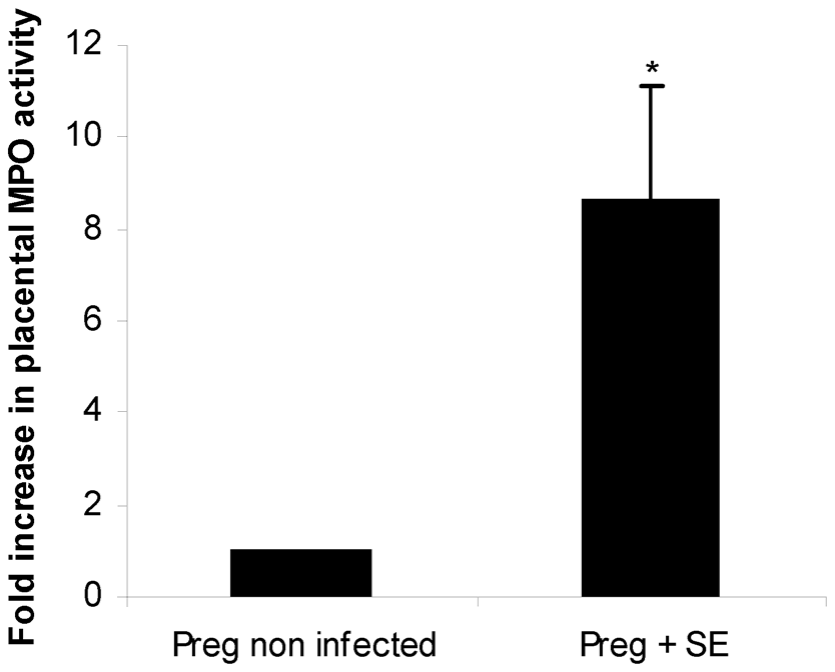
MPO activity in placenta after *S.* Enteritidis enterocolitis. Pregnant mice received 20 mg of streptomycin 24 h before intragastric infection with 3–4×10^3^ CFU of *S.* Enteritidis (day 15 of gestation). A group of non infected pregnant mice was included as control. Placentas (healthy and infected) were obtained on day 18 of gestation. MPO assay was performed as described in materials and methods. Data are presented as fold increase relative to non infected control, set as 1. Placentas from each individual mouse were pooled. Five animals per group were analyzed. Results are expressed as mean +/− SD. *p<0.01 respect to uninfected control placentas. Representative data from 3 independent experiments.

**Figure 4:**
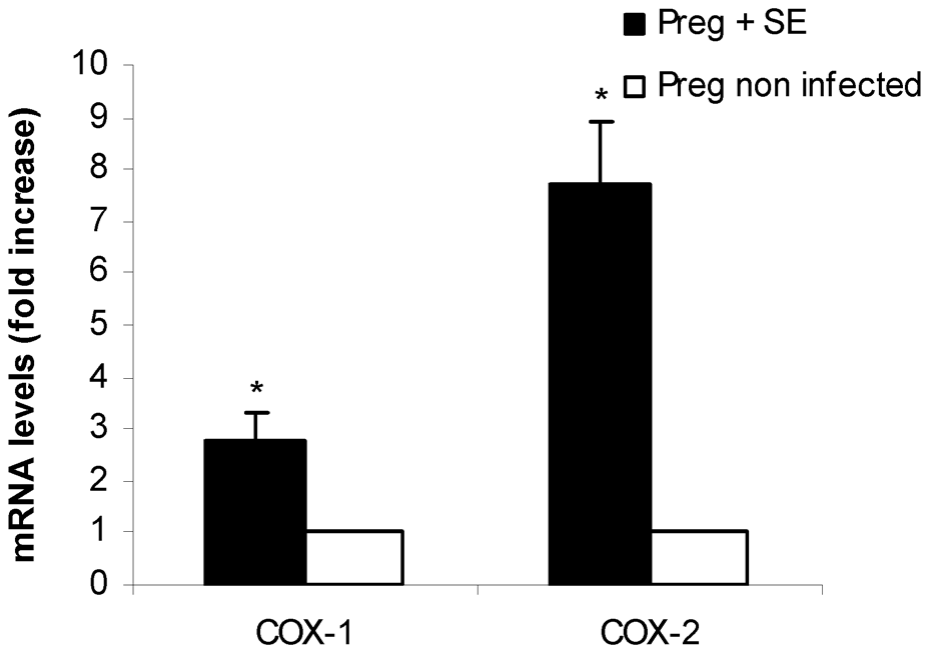
Placental COX-1 and COX-2 expression after *S.* Enteritidis enterocolitis. Pregnant mice received 20 mg of streptomycin 24 h before intragastric infection with 3–4×10^3^ CFU of *S.* Enteritidis (day 15 of gestation). A group of non infected pregnant mice was included as control. Placentas (healthy and infected) were obtained on day 18 of gestation. The relative COX-1 and COX-2 mRNA amount was determined by qPCR and related to mRNA levels in non infected control, set as 1. Placentas from each individual mouse were pooled. Five animals per group were analyzed. Results are expressed as mean +/− SD. *p<0.05 respect to uninfected control placentas. Representative data from 3 independent experiments.

All fetuses delivered throughout the experiments (i.e.: at term, pre term, naturally or by cesarean section) were included in this analysis. Fetuses were weighted and FGR was determined as described in materials and methods. We found no adverse effects of streptomycin treatment on the average fetal weight of pups born at term from uninfected mice. In contrast, significant differences (p<0.001) were observed in the weight of pups born from infected mothers (either delivered pre term or at term) compared with their corresponding controls. As shown in Table 5, pups from infected mothers weighed almost 50% less than uninfected controls (0.815 g vs. 1.543 g for those delivered at term, and 0.622 g vs. 1.231 g for pre term, respectively). These results indicate that *Salmonella* enterocolitis during late stages of pregnancy induces FGR. It is important to note that all fetuses in the litter were low weighted. FGR is a pathological condition usually associated to low oxygen transport across the placenta [23]. It is likely that, in our model, placental hypoxia takes place during *Salmonella* infection because of cellular infiltration. Thus, we analyzed cellular infiltration and hypoxia markers in placenta from infected and uninfected pregnant mice. MPO activity, the marker for cellular infiltration, was higher (p<0.01) in the *S.* Enteritidis-infected placenta as opposed to the uninfected pregnant host (Figure 3). This finding is compatible with an influx of inflammatory cells towards the placenta triggered by *Salmonella* enterocolitis. Next, hypoxia markers COX-1 and COX-2 (proposed by Agudelo et al [24]) were investigated by qPCR. As shown in Figure 4, COX-1 and COX-2 levels were significantly higher in infected placentas than in healthy ones (p<0.05). Altogether these results suggest that FGR observed in the litter from *Salmonella-*infected mothers is due, in part, to placental hypoxia. Because hypoxia is a known trigger of apoptosis in different tissues we decided to investigate whether *Salmonella* infection was inducing apoptosis in placenta.

**Table 5:** Average fetal weight in *S.* Enteritidis infected and noninfected mothers.

Fetal growth	Dose of streptomycin	Dose	Term delivery	Pre term delivery
	mg/mice	CFU/mice	mean fetal ± SD (n) (g)	mean fetal ± SD (n) (g)
FGR(a)	20	10^3^	0.815±0.078* (66)	0.622±0.126* (61)
NFW(b)	0	0	1.543±0.061 (134)	1.231±0.091 (84)
NFW(b)	20	0	1.499±0.052 (31)	ND(c)

Pregnant mice received 20 mg of streptomycin 24 h before intragastric infection with 3–4×10^3^ CFU of *S.* Enteritidis (day 15 of gestation). Uninfected pregnant mice (with or without streptomycin treatment) were included as controls. One group of animals was allowed to deliver normally and the other group was sacrificed at 3 days post infection (day 18 of gestation). Fetuses were weighted and FGR was calculated as described in materials and methods.

(a) FGR, Fetal Growth Restriction.

(b) NFG, Normal Fetal Weight.

(c) ND, Not determined.

*p<0.001 respect to uninfected control fetuses.

### Enterocolitis by *S.* Enteritidis induces apoptosis in placental tissue

**Figure 5:**
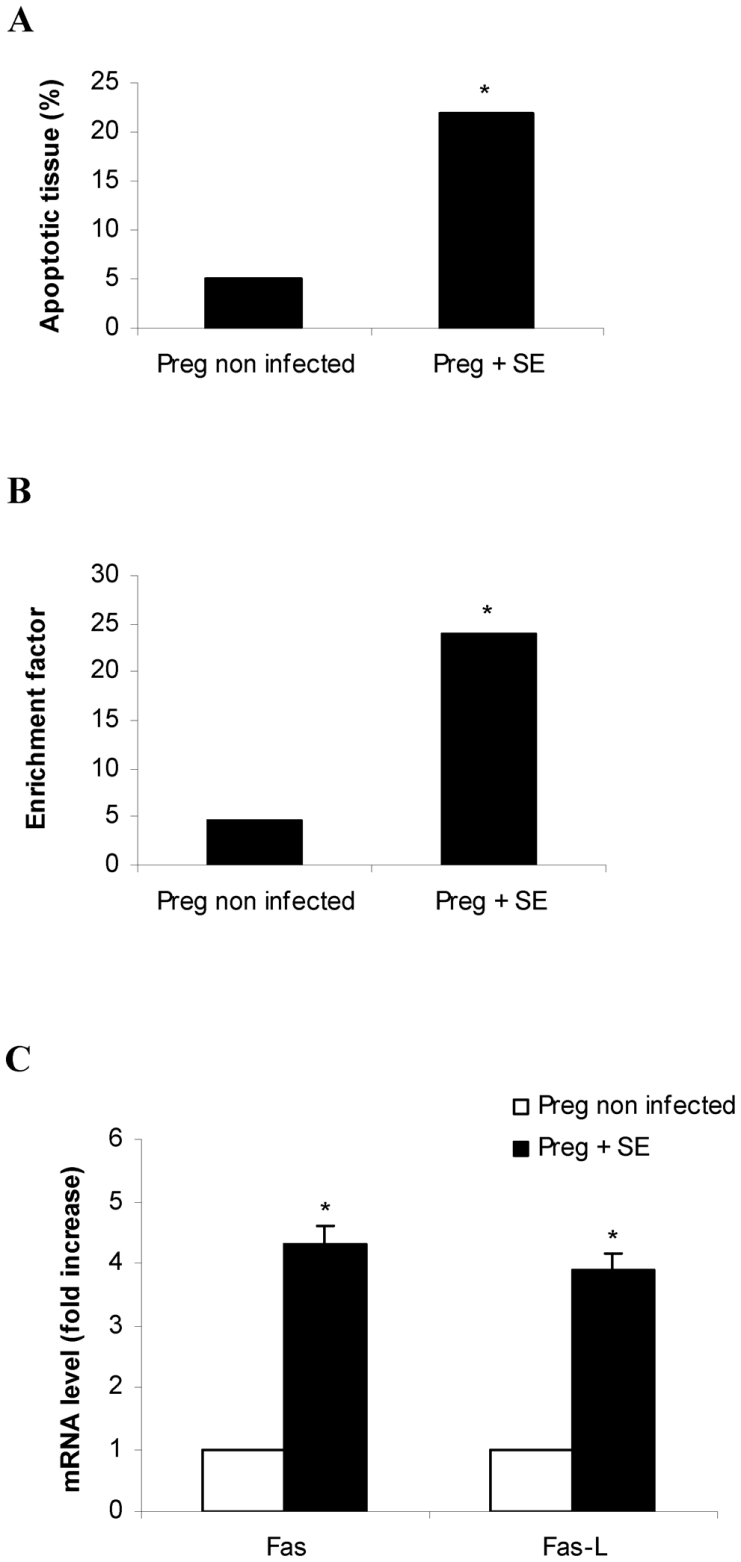
Placental apoptosis after *S.* Enteritidis enterocolitis. Pregnant mice received 20 mg of streptomycin 24 h before intragastric infection with 3–4×10^3^ CFU of *S.* Enteritidis (day 15 of gestation). A group of non infected pregnant mice was included as control. Placentas (healthy and infected) were obtained on day 18 of gestation. **A.** Apoptotic cells were counted in hematoxylin-eosin-stained sections using the morphometric procedures described in materials and methods. **B.** Apoptosis was assessed using a cell death detection ELISA. Placentas from each individual mouse were pooled. The *y* axis represents DNA fragmentation, as measured by the enrichment factor as described in materials and methods. Results are expressed as median. *p<0.05 respect to uninfected control placentas. Representative data from 3 independent experiments. **C.** The relative Fas and Fas-L mRNA amount was determined by qPCR and related to mRNA levels in non infected control, set as 1. Placentas from individual mice were pooled. Five animals per group were analyzed. Results are expressed as mean +/− SD. *p<0.05 respect to uninfected control placentas. Representative data from 3 independent experiments.

Apoptosis was investigated utilizing a morphometric procedure, analyzing DNA fragmentation by ELISA and measuring Fas and Fas-L expression by qPCR. Placentas (healthy and infected) delivered by cesarean on day 18 of gestation were included in this study. Apoptotic cells were counted in hematoxylin-eosin-stained sections as described in materials and methods (Figure 5A). Results showed that the percentage of apoptotic tissue in *Salmonella* infected placentas (median, 22%; range, 19 to 24%) was significantly higher (p<0.05) than that found in control mice (median, 5%; range, 3 to 6%). Similar results were obtained when DNA fragmentation was assessed in the placental tissue from pregnant mice infected with *S.* Enteritidis (Figure 5B). Again, statistically significant differences (p<0.05) were found in infected mothers compared with the control group. In the next series of experiments we examined the expression of Fas and Fas-L mRNA in the placentas infected with *Salmonella.* qPCR assay revealed that Fas and Fas-L mRNA expression is higher (ca. 4-fold increase; p<0.05) in the infected placentas than in control ones (Figure 5C). These observations implicate apoptosis as a host response or a downstream effect of *S.* Enteritidis. Taken together, our findings strongly suggest that *S.* Enteritidis enterocolitis during late stages of pregnancy leads to placental dysfunction (hypoxia, cellular infiltration and apoptosis) that contributes to the impairment of intrauterine fetal development.

## Discussion

Food- and waterborne diseases are on the rise, and these can get exacerbated in immunocompromised hosts. Pregnancy does not result in compromised immunity against all microorganisms; however, it is not clear why some pathogens, including *Salmonella*, pose an increased risk [25]. The relationship between pregnancy, pathogen virulence, pathogen burden, inflammation, and the consequent pregnancy outcome needs further investigation. Here we show, using a self-limiting enterocolitis model, that the ingestion of a low dose of *S.* Enteritidis is sufficient to induce massive infection of placenta, uterus and fetuses.

Unlike the non-pregnant enterocolitis model, in which the disease is self-limiting [26], [27], the ingestion of a low dose of *S.* Enteritidis during late stages of pregnancy resulted in a disseminated infection. Oral *Salmonella* infection of streptomycin-pretreated mice has been widely utilized to study the early stages of enterocolitis [12], [28,]. Very recently, it was shown that a single dose of streptomycin induces low level of intestinal inflammation characterized by cellular infiltration [29,]. Therefore, it would be reasonable to infer that streptomycin “per se” contributes to the inflammatory response described here in pregnant infected mice. We found however, that a single dose of streptomycin does not modify the outcome of pregnancy. Moreover, in a previous work we showed that the expression of intestinal inflammatory cytokines, such as IL-17, IL-23, IL-1β, IL-6 and TNF-α is not increased in streptomycin-treated mice [27]. Consequently, it seems clear that the deleterious effect of *Salmonella* enterocolitis during late stages of pregnancy shown in this work cannot be attributed to streptomycin treatment.

The increased bacterial translocation that occurs during pregnancy [30] could explain the higher bacterial burden in Peyer's patches and spleen found in pregnant mice compared with non-gravid controls. Placenta, uterus and fetuses also showed a substantial bacterial colonization 3 days after oral infection. This finding is in agreement with the ability of *S.* Typhimurium to rapidly proliferate in the secluded placental environment [19]. Placental cells allow profound intracellular proliferation of *S.* Typhimurium [25] and trophoblast cells actively uptake *Salmonella* by receptor-mediated endocytosis [31].

The high bacterial burden in the placenta could be in line with the increased placental expression of IL-10 produced. It has been shown that IL-10 production by trophoblasts prevents *Salmonella*-Containing Vacuole maturation culminating in profound bacterial proliferation with early phagosomes. In fact, trophoblast cells provide a safe early endosomal niche for *Salmonella* to multiply under conditions of minimum stress [31]. We observed that spleen is also highly colonized in pregnant mice (ca. 10.000-fold increase compared with the non gravid host), most likely as a result of the reverse trafficking of *Salmonella* from placenta. Similarly, Pejcic-Karapetrovic et al observed that the increase in splenic bacteria is preceded by heavy placental infection [19]. More recently, it has been proposed that trophoblast cells can act as a reservoir for *Salmonella* dissemination in the pregnant host [31]. Rowe et al demonstrated that IL-10 is dispensable for sustaining pregnancy but impairs host defense against prenatal *Listeria monocytogenes* infection. These authors suggest that IL-10 neutralization may be used for boosting immunity against infection without compromising pregnancy outcomes [32].

We found that ingestion of *S.* Enteritidis during late stages of pregnancy induces premature labor and abortion. This is not surprising since infections and associated inflammation are considered to play a pivotal role in premature birth [33], [34], spontaneous abortion, fetal growth restriction [35] and placental pathologies [36], [37]. Moreover, inflammatory cytokines such as IFN-γ, TNF-α, and IL-12 can induce resorptions and are up-regulated in pre term labor [38]. Likewise, we found that at day 18 of gestation -when the majority of pre term deliveries took place- *Salmonella* infection increases the expression of inflammatory cytokines in placenta, amniotic fluid and maternal serum. In our model of infection, the increase in IL-17 induced by *Salmonella* might be disadvantageous for the maintenance of pregnancy. Saito et al found that Th17 cells promote inflammation at the feto–maternal interface in pre term delivery [39]. Here we show that, concomitantly with augmented levels of IL-17, *Salmonella* infected placenta presented elevated levels of MPO and IFN-γ. This is in line with the role of IL-17 in neutrophil infiltration and with the fact that neutrophils are the main source of early IFN-γ production. It is likely that cellular infiltration originated as a response to *Salmonella* infection could affect oxygen transport across the placenta and contribute to fetal growth restriction. In fact, some hypoxia mechanisms including oxygen consumption by infiltrating cells, decrease blood perfusion and reduce the effective fetal-maternal surface area [40]. Indeed, we found that hypoxia markers (COX-1 and COX-2) were elevated in *Salmonella-*infected placenta. A recent report showed that during placental malaria there is an increase in the expression of COX-2 and IL-10 [24], [41]. To our knowledge this is the first time that an increase in hypoxia markers is associated to placental infection with *Salmonella*. As an etiopathologic factor, hypoxia is a known trigger of apoptosis in different tissues. Therefore, placenta may respond to hypoxic stress by enhancing the number of apoptotic cells [42]. Certainly, we found that *Salmonella* infection during pregnancy induces apoptosis in placental tissue, affecting intrauterine fetuses' development. We observed an increment of apoptotic cells in infected placental tissue respect to the control. This result is correlated with an increase in mRNA expression of Fas FasL system in *Salmonella*-infected placenta vs. control. Fas and FasL expression have been proposed as markers of apoptosis, and this is considered an important mechanism by which cytokines act locally and may influence critical signaling processes [24]. In other pathologies such as preeclampsia, the high rate of apoptosis has been directly linked with intrauterine growth restriction [43]. Results presented here strongly suggest that *Salmonella* infection during late stages of pregnancy leads to placental apoptosis affecting fetal growth.

In summary, the ingestion of a low dose of *S.* Enteritidis resulted in massive infection during late stages of pregnancy leading to placental infection, cellular infiltration, hypoxia, apoptosis and FGR. The inflammatory cytokine response to *Salmonella* was evident not only in placenta, but also in amniotic fluid and maternal serum contributing to a detrimental effect on pregnancy outcome. The loss of pregnancy by salmonellosis is poorly documented. However, our study highlights the elevated risk of pregnant mothers to suffering pregnancy complications, given the increasing incidence of such food-borne infections. Our model provides a suitable reproducible tool to interpret the role of infections and inflammation during pregnancy. This opens up further opportunities for deeper thoughts in vaccine development, management and treatment.

## References

[1] Bennett SD, Manikonda K, Mungai E, Dewey-Mattia DL, Gould LH (2012) Surveillance for Foodborne Disease Outbreaks United States Annual Report. Available: http://www.cdc.gov/foodsafety/pdfs/foodborne-disease-outbreaks-annual-report-2012-508c.pdf. Accessed 2014 May 3.

[2] Mastroeni P (2002) Immunity to systemic *Salmonella* infections. Curr Mol Med 2: 393–406. 12108950 10.2174/1566524023362492

[3] Jamieson DJ, Theiler RN, Rasmussen SA (2006) Emerging infections and pregnancy. Emerg Infect Dis 12: 1638–43. 17283611 10.3201/eid1211.060152 PMC3372330

[4] Dunn PL, North RJ (1995) Virulence ranking of some *Mycobacterium tuberculosis* and *Mycobacterium bovis* strains according to their ability to multiply in the lungs, induce lung pathology, and cause mortality in mice. Infect Immun 63: 3428–3437. 7642273 10.1128/iai.63.9.3428-3437.1995 PMC173472

[5] Negi VD, Nagarajan AG, Chakravortty D (2010) A safe vaccine (DV-STM-07) against *Salmonella* infection prevents abortion and confers protective immunity to the pregnant and new born mice. PLoS One 10: e9139 10.1371/journal.pone.0009139 PMC2818715 20161765

[6] Fievet N, Moussa M, Tami G, Maubert B, Cot M, et al (2001) *Plasmodium falciparum* induces a Th1/Th2 disequilibrium, favoring the Th1-type pathway, in the human placenta. J Infect Dis 183: 1530–1534. 11319691 10.1086/320201

[7] Krishnan L, Guilbert LJ, Russell AS, Wegmann TG, Mosmann TR, et al (1996) Pregnancy impairs resistance of C57BL/6 mice to Leishmania major infection and causes decreased antigen-specific IFN-gamma response and increased production of T helper 2 cytokines. J Immunol 156: 644–652. 8543816

[8] Luft BJ, Remington JS (1982) Effect of pregnancy on resistance to *Listeria monocytogenes* and *Toxoplasma gondii* infections in mice. Infect Immun 38: 1164–1171. 6818146 10.1128/iai.38.3.1164-1171.1982 PMC347871

[9] Awadalla SG, Mercer LJ, Brown LG (1985) Pregnancy complicated by intraamniotic infection by *Salmonella typhi* . Obstet Gynecol 65: 30S–31S. 3871929

[10] Hedriana HL, Mitchell JL, Williams SB (1995) *Salmonella typhi* chorioamnionitis in a human immunodeficiency virus-infected pregnant woman. A case report. J Reprod Med 40: 157–159. 7738931

[11] Schloesser RL, Schaefer V, Groll AH (2004) Fatal transplacental infection with non-typhoidal *Salmonella* . Scand J Infect Dis 36: 773–774. 15513410 10.1080/00365540410020802

[12] Hapfelmeier S, Hardt W (2005) A Mouse Model for S. typhimurium-Induced Enterocolitis. Trends Microbiology 13: 497–503. 10.1016/j.tim.2005.08.008 16140013

[13] Lin D, Smith MA, Elter J, Champagne C, Downey CL, et al (2003) *Porphyromonas gingivalis* infection in pregnant mice is associated with placental dissemination, an increase in the placental Th1/Th2 cytokine ratio, and fetal growth restriction. Infect Immun 71: 5163–8. 12933860 10.1128/IAI.71.9.5163-5168.2003 PMC187373

[14] Bradley PP, Priebat DA, Christensen RD, Rothstein G (1982) Measurement of cutaneous inflammation: estimation of neutrophil content with an enzyme marker. J Invest Dermatol 78: 206–209. 6276474 10.1111/1523-1747.ep12506462

[15] Font-Nieves M, Sans-Fons MG, Gorina R, Bonfill-Teixidor E, Salas-Pérdomo, et al (2012) Induction of COX-2 enzyme and down-regulation of COX-1 expression by lipopolysaccharide (LPS) control prostaglandin E2 production in astrocytes. J Biol Chem 24: 6454–68. 10.1074/jbc.M111.327874 PMC3307308 22219191

[16] Cerquetti MC, Hovsepian E, Sarnacki SH, Goren NB (2008) *Salmonella enterica* serovar Enteritidis *dam* mutant induces low NOS-2 and COX-2 expression in macrophages via attenuation of MAPK and NF-kappaB pathways. Microbes Infect 10: 1431–9. 18801455 10.1016/j.micinf.2008.08.008

[17] Hovsepian E, Penas F, Goren NB (2010) 15-deoxy-Δprostaglandin GJ2 but not rosiglitazone regulates metalloproteinase 9, Nos-2, and ciclooxygenase 2 expression and functions by peroxisome proliferators activated receptor γ-dependent and independent mechanisms in cardiac cells. Shock 34: 60–67. 19997048 10.1097/SHK.0b013e3181cdc398

[18] Godinez I, Raffatellu M, Chu H, Paixão TA, Haneda T, et al (2009) Interleukin-23 Orchestrates Mucosal Responses to *Salmonella enterica* serotype Typhimurium in the Intestine. Infect Immun 1: 387–398. 10.1128/IAI.00933-08 PMC2612270 18955477

[19] Pejcic-Karapetrovic B, Gurnani K, Russell MS, Finlay BB, Sad S, et al (2007) Pregnancy impairs the innate immune resistance to *Salmonella typhimurium* leading to rapid fatal infection. J Immunol 179: 6088–96. 17947683 10.4049/jimmunol.179.9.6088

[20] Kusakabe K, Otsuki Y, Kiso Y (2005) Involvement of the fas ligand and fas system in apoptosis induction of mouse uterine natural killer cells. J Reprod Dev 51: 333–40. 15781991 10.1262/jrd.16086

[21] Lee BP, Mansfield E, Hsieh SC, Hernandez-Boussard T, Chen W, et al (2005) Expression profiling of murine double-negative regulatory T cells suggest mechanisms for prolonged cardiac allograft survival. J Immunol. 174: 4535–44. 10.4049/jimmunol.174.8.4535 15814674

[22] Zhou HR, Harkema JR, Hotchkiss JA, Yang D, Roth RA, et al (1999) Lipopolysaccharide and the trichothecene vomitoxin (deoxynivalenol) synergistically induce apoptosis in murine lymphoid organs. Toxicol Sci 53: 253–263. 10.1093/toxsci/53.2.253 10696773

[23] Whitehead CL, Tong S (2014) Measuring hypoxia-induced RNA in maternal blood: a new way to identify critically hypoxic fetuses in utero? Expert Rev Mol Diagn 14: 509–11 10.1586/14737159.2014.915749 24779397

[24] Agudelo OM, Aristizabal BH, Yanow SK, Arango E, Carmona-Fonseca J, et al (2014) Submicroscopic infection of placenta by *Plasmodium* produces Th1/Th2 cytokine imbalance, inflammation and hypoxia in women from north-west Colombia. Malar J 13: 2–9 10.1186/1475-2875-13-122 24673747 PMC3972514

[25] Chattopadhyay A, Robinson N, Sandhu JK, Finlay BB, Sad S, et al (2010) *Salmonella enterica* serovar Typhimurium-induced placental inflammation and not bacterial burden correlates with pathology and fatal maternal disease. Infect Immun 78: 2292–2301 10.1128/IAI.01186-09 20194592 PMC2863547

[26] Noto Llana M, Sarnacki SH, Vázquez MV, Gartner AS, Giacomodonato MN, et al (2012) *Salmonella enterica* induces joint inflammation and the expression of IL-17 in draining lymph nodes early after the onset of enterocolitis in mice. Infect Immun 6: 2231. 10.1128/IAI.00324-12 PMC3370572 22493084

[27] Noto Llana M, Sarnacki SH, Aya Castañeda M del R, Bernal MI, Giacomodonato MN, et al (2013) Consumption of *Lactobacillus casei* fermented milk prevents *Salmonella* reactive arthritis by modulating IL-23/IL-17 expression. PLoS One 8: e82588 10.1371/journal.pone.0082588 24340048 PMC3858332

[28] Barthel M, Hapfelmeier S, Quintanilla-Martínez L, Kremer M, Rohde M, et al (2003) Pretreatment of mice with streptomycin provides a *Salmonella enterica* serovar Typhimurium colitis model that allows analysis of both pathogen and host. Infect Immun 71: 2839–2858 10.1128/IAI.71.5.2839-2858.2003 12704158 PMC153285

[29] Spees AM, Wangdi T, Lopez CA, Kingsbury DD, Xavier MN, et al (2013) Streptomycin-induced inflammation enhances *Escherichia coli* gut colonization through nitrate respiration. MBio 4: e00430–13 10.1128/mBio.00430-13 PMC3705454 23820397

[30] Donnet-Hughes A, Perez PF, Doré J, Leclerc M, Levenez F, et al (2010) Potential role of the intestinal microbiota of the mother in neonatal immune education. Proc Nutr Soc 69: 407–15 10.1017/S0029665110001898 20633308

[31] Nguyen T, Robinson N, Allison SE, Coombes BK, Sad S, et al (2013) IL-10 produced by trophoblast cells inhibits phagosome maturation leading to profound intracellular proliferation of *Salmonella enterica* Typhimurium. Placenta 34: 765–74 10.1016/j.placenta.2013.06.003 23834952 PMC3797447

[32] Rowe JH, Ertelt JM, Aguilera MN, Farrar MA, Way SS (2011) Foxp3(+) regulatory T cell expansion required for sustaining pregnancy compromises host defense against prenatal bacterial pathogens. Cell Host Microbe 10: 54–64 10.1016/j.chom.2011.06.005 21767812 PMC3140139

[33] Goldenberg RL, Hauth JC, Andrews WW (2000) Intrauterine infection and pre term delivery. N Engl J Med 342: 1500–1507. 10816189 10.1056/NEJM200005183422007

[34] Redline RW (2004) Placental inflammation. Semin Neonatol 9: 265–274. 15251143 10.1016/j.siny.2003.09.005

[35] Challis JR, Lockwood CJ, Myatt L, Norman JE, Strauss JF 3rd, et al (2009) Inflammation and pregnancy. Reprod Sci 16: 206–215. 19208789 10.1177/1933719108329095

[36] Conrad KP, Miles TM, Benyo DF (1998) Circulating levels of immunoreactive cytokines in women with preeclampsia. Am J Reprod Immunol 40: 102–111. 9764352 10.1111/j.1600-0897.1998.tb00398.x

[37] Saji F, Samejima Y, Kamiura S, Sawai K, Shimoya K, et al (2000) Cytokine production in chorioamnionitis. J Reprod Immunol 47: 185–196. 10924750 10.1016/s0165-0378(00)00064-4

[38] Chaouat G, Menu E, Clark DA, Dy M, Minkowski M, et al (1990) Control of fetal survival in CBA × DBA/2 mice by lymphokine therapy. J Reprod Fertil 89: 447–458. 2119428 10.1530/jrf.0.0890447

[39] Saito S, Nakashima A, Shima T, Ito M (2010) Th1/Th2/Th17 and regulatory T-cell paradigm in pregnancy. Am J Reprod Immunol 63: 601–610. 20455873 10.1111/j.1600-0897.2010.00852.x

[40] Takem EN, D'Alessandro U (2013) Malaria in pregnancy. Mediterr J Hematol Infect Dis 5: e2013010. 23350023 10.4084/MJHID.2013.010 PMC3552837

[41] Sarr D, Aldebert D, Marrama L, Frealle E, Gaye A, et al (2010) Chronic infection during placental malaria is associated with up-regulation of cycloxygenase-2. Malar J 9: 2–9. 20144201 10.1186/1475-2875-9-45 PMC2831904

[42] Erel CT, Dane B, Calay Z, Kaleli S, Aydinli K (2001) Apoptosis in the placenta of pregnancies complicated with IUGR. Int J Gynaecol Obstet 73: 229–235. 11376669 10.1016/s0020-7292(01)00373-3

[43] Guller S, Ma YY, Fu HH, Krikun G, Abrahams VM (2008) The placental syncytium and the pathophysiology of preeclampsia and intrauterine growth restriction: a novel assay to assess syncytial protein expression. Ann N Y Acad Sci 1127: 129–133. 18443340 10.1196/annals.1434.015 PMC3671376

